# Magnetic Resonance Imaging at 7T Reveals Common Events in Age-Related Sarcopenia and in the Homeostatic Response to Muscle Sterile Injury

**DOI:** 10.1371/journal.pone.0059308

**Published:** 2013-03-12

**Authors:** Antonio Esposito, Lara Campana, Anna Palmisano, Francesco De Cobelli, Tamara Canu, Francesco Santarella, Caterina Colantoni, Antonella Monno, Michela Vezzoli, Giulio Pezzetti, Angelo A. Manfredi, Patrizia Rovere-Querini, Alessandro Del Maschio

**Affiliations:** 1 Department of Radiology and Preclinical MR and US Facility of Experimental Imaging Center, San Raffaele Scientific Institute, Milano, Italy; 2 Vita-Salute San Raffaele University, Milano, Italy; 3 Innate Immunity and Tissue Remodeling Unit, Division of Regenerative Medicine, Stem Cell and Gene Therapy, San Raffaele Scientific Institute, Milano, Italy; 4 Autoimmunity and Vascular Inflammation Unit, Division of Regenerative Medicine, Stem Cell and Gene Therapy, San Raffaele Scientific Institute, Milano, Italy; University of Rome La Sapienza, Italy

## Abstract

Skeletal muscle remodeling in response to various noxae physiologically includes structural changes and inflammatory events. The possibility to study those phenomena *in-vivo* has been hampered by the lack of validated imaging tools. In our study, we have relied on multiparametric magnetic resonance imaging for quantitative monitoring of muscle changes in mice experiencing age-related sarcopenia or active regeneration after sterile acute injury of tibialis anterior muscle induced by cardiotoxin (CTX) injection. The extent of myofibrils’ necrosis, leukocyte infiltration, and regeneration have been evaluated and compared with parameters from magnetic resonance imaging: T2-mapping (T2 relaxation time; T2-rt), diffusion-tensor imaging (fractional anisotropy, F.A.) and diffusion weighted imaging (apparent diffusion coefficient, ADC). Inflammatory leukocytes within the perimysium and heterogeneous size of fibers characterized aged muscles. They displayed significantly increased T2-rt (P<0.05) and F.A. (P<0.05) compared with young muscles. After acute damage T2-rt increased in otherwise healthy young muscles with a peak at day 3, followed by a progressive decrease to basal values. F.A. dropped 24 hours after injury and afterward increased above the basal level in the regenerated muscle (from day 7 to day 15) returning to the basal value at the end of the follow up period. The ADC displayed opposite kinetics. T2-rt positively correlated with the number of infiltrating leucocytes retrieved by immunomagnetic bead sorting from the tissue (r = 0.92) and with the damage/infiltration score (r = 0.88) while F.A. correlated with the extent of tissue regeneration evaluated at various time points after injury (r = 0.88). Our results indicate that multiparametric MRI is a sensitive and informative tool for monitoring inflammatory and structural muscle changes in living experimental animals; particularly, it allows identifying the increase of T2-rt and F.A. as common events reflecting inflammatory infiltration and muscle regeneration in the transient response of the tissue to acute injury and in the persistent adaptation to aging.

## Introduction

A non-invasive tool for the quantitative and dynamic assessment of the skeletal muscle modifications, during degenerative conditions or acute damage and regeneration, would be a valuable tool in both the clinical and preclinical settings. Magnetic Resonance Imaging (MRI) is a promising tool, which could theoretically provide information on the changes associated to skeletal muscle injury, such as the inflammatory response and the regenerative events. A fine characterization of the link between imaging parameters and the events occurring in the tissue would make the interpretation of imaging data easier and more straightforward in preclinical settings.

In particular, Magnetic Resonance Diffusion Tensor Imaging (DTI) allows to investigate the skeletal muscle architecture assessing the diffusion of water molecules in diverse directions of space [1]. Due to the fibrillar structure of skeletal muscle water diffusion is greater along fibers orientation than in other directions. This anisotropic diffusion that characterizes intact skeletal muscles is quantified with relative ease by the Epi-DTI sequence, measuring the fractional anisotropy (F.A.) [2].

During acute injuries or significant tissue degeneration the anisotropic property may be disrupted, with the loss of the preferential direction of water molecules diffusion. Accordingly, a decrease of F.A. after an experimental ischemic injury and a progressive F.A. increase during regenerative phases was described [3]. An increased signal in T2-weighted images linked to T2 relaxation-time (T2-rt) lengthening is a commonly accepted MRI sign of muscle damage, which is supposed to reflect heterogeneous phenomena as muscle edema, inflammation and necrosis [4], [5], [6], [7], [8].

So far, the correlation between the MRI parameters and the dynamic events taking place during skeletal muscle damage and repair was not extensively characterized. Moreover very little is known about the events taking place in the aging muscle. Loss of muscle mass and strength is a well-recognized part of muscle aging and is associated to fragility and functional decline. This process has been originally considered as a merely degenerative event. However, more recent studies have clearly implicated inflammatory and immunological cues that restrict the ability of muscle stem cells both to repair the tissue, thus preventing age-associated wasting, and to maintain intact the size of their own pool [9]. However, the impact of inflammation on sarcopenia is difficult to unambiguously assess, particularly when sarcopenia is at its onset. In our study, we prospectively assessed a multi-parametric MRI protocol as a non-invasive and quantitative tool to assess muscle wasting conditions, injury, and healing processes in experimental mice, by correlating the imaging results with histological information on the extent of inflammatory infiltrates and fiber regeneration. Our results demonstrate that MRI is a valuable tool to analytically and non-invasively obtain information on ongoing skeletal muscle damage and healing *in vivo*, allowing to finely discriminate in parallel the extent of inflammation and regeneration.

## Materials and Methods

### Ethics statement

All experiments were carried out in strict accordance with protocols approved by the San Raffaele Scientific Institute (Milan, Italy) Institutional Animal Care and Use Committee (Permit numbers 371 and 512). Mice were evaluated for physical health during the entire period of follow-up. In particular body weight and kyphosis were evaluated on a daily basis and mice were sacrificed before the end of the experiment in case of weight loss higher than 20% and/or occurrence of marked kyphosis. These events never occurred in our study, hence no mice were prematurely sacrificed. At the end of the experiment mice were sacrificed using a CO_2_ chamber to minimize animal suffering.

### Animal model and study design

**Figure 1:**
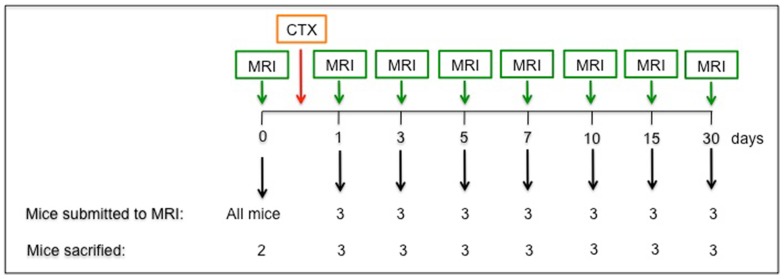
Study design. Schematic representation of the experimental design of the longitudinal study realized in acutely injured mice.

The tibialis anterior (TA) muscle of 23 C57BL/6 mice (Charles River) were injured by injection of cardiotoxin (CTX, 15 μM) (*Naja mossambica mossambica*, Sigma-Aldrich), a snake venom that selectively injures myofibers by disturbing calcium homeostasis at the neuromuscular junction, followed by necrosis of muscle fibers [10]. The non-injured gastrocnemius (G) muscle was used as an internal control. MRI data and corresponding histology were assessed both before injury (n = 2) and at various time points after CTX injection (1, 3, 5, 7, 10, 15 and 30 days after; n = 3 at each time-point): at each time point animals underwent MRI and were sacrificed immediately after imaging (Figure 1). Injured and non-injured muscles were collected, frozen and stained for histological analysis. A cohort of four mice was monitored with MRI along the entire healing process (time points: before and 1, 3, 7, 10, 15 and 30 days after damage) and sacrificed at day 30; muscles were collected and processed as above. MRI imaging and histological features were also analyzed in independent experiments in 14 mice to evaluate age-associated sarcopenia comparing young (two months old) and old (eighteen months old) C57BL/6 mice.

### MR Imaging

MRI studies were performed on a 7T preclinical magnetic resonance scanner (Bruker, BioSpec 70/30 USR, Paravision 5.0, Germany), equipped with 450/675 mT/m gradients (slew-rate: 3400–4500T/m/s; rise-time: 140µs). A phased-array rat-heart coil with four internal preamplifiers was used as receiver, coupled with a 72 mm linear-volume coil as transmitter. Mice were under general anesthesia obtained by 1,5–2% isoflurane (Forane®, Abbott) vaporized in 100% oxygen (flow: 1l/min), in prone position, with the right leg fixed in the center of the coil. Breathing and body temperature were monitored during MRI (SA Instruments, Inc., Stony Brook, NY, USA) and maintained around 30 breaths-per-minute and 37°C, respectively. After positioning in the magnet isocenter, a fieldmap based shimming (MAPSHIM software package, ParaVision-5.0, Bruker; Germany) was performed to optimize B0 field homogeneity. MRI protocol included T2-mapping, diffusion-mapping, and DTI. Muscle T2-maps were obtained using a Multi-Slice-Multi-Echo (MSME) sequence with fat suppression (repetition-time = 1938 ms; 16 echo-times = 10.73/171.68 ms; field-of-view = 20×20 mm; matrix = 256×256; spatial resolution = 0.078×0.078 mm/pixel; NSA = 4) acquired on axial plane (10 slices; thickness = 1 mm; gap = 0 mm). An EPI diffusion weighted (DWI) sequence with 6 b-values (100-200-400-600-800-1000 s/mm2) was employed to calculate the apparent diffusion coefficient (ADC) map (repetition-time = 3000 ms; echo-time = 30 ms; diffusion gradient duration = 7 ms; diffusion gradient separation = 14 ms; NSA = 2). Diffusion tensor images were obtained using a SpinEcho-EPI sequence (DTI-Epi) with 30 diffusion gradient directions (repetition-time = 3750 ms; echo-time = 33 ms; b-values for direction = 0 sec/mm2-700 sec/mm2; diffusion gradient duration = 4 ms; diffusion gradient separation = 20 ms; NSA = 2). DWI and DTI-Epi sequence shared the same geometrical features (field-of-view = 30×30 mm; matrix = 128×128; spatial resolution = 0.234×0.234 mm/pixel; 10 slices; slice thickness = 1 mm; gap = 0 mm).

### Image analysis

MRI post-processing was performed with Paravision-5.0 software (Bruker). Average ADC, F.A. and T2-rt values were obtained from the regions-of-interest (ROIs) of five subsequent slices placed both on tibialis anterior (damaged muscle) and on gastrocnemius (non-injured muscle) muscles of each mouse at each time point.

### Histological analysis

7μm thick muscle sections were stained by haematoxylin-eosin (H&E, BioOptica). For each muscle, four serial sections were collected and stained. An expert blinded pathologist evaluated each sample assigning a 0-to-5 score (0: healthy muscles; 5: maximum infiltration) based on the histological degree of muscle infiltration after injury. This damage/infiltration score was correlated with T2-rt. A second independent blinded pathologist counted regenerating center-nucleated fibers to obtain a semi-quantitative assessment of the extent of muscle regeneration. The percentage of center-nucleated fibers was correlated with F.A. measured by the DTI sequence. In the sections of old *vs.* young mice we quantified the number of fibers/field of vision (FOV) using 20x magnification of H&E stained sections. 5 animals/group were evaluated. On the same sections we have measured the cross sectional area (c.s.a.) of the fibers to evaluate dimensions, as described [11], [12].

To unambiguously assess the extent of muscle infiltration by inflammatory cells, leukocytes were identified as CD45^+^ cells among mononucleated cells isolated from enzymatically-digested muscles and retrieved by immune-magnetic cell sorting, as described [13]. Briefly, muscles were digested with collagenase (Sigma-Aldrich) and dispase (Gibco). Cells were labeled using magnetic beads linked to anti-mouse CD45-antibody (Miltenyi-Biotech) and passed through a MS magnetic column (Miltenyi-Biotech). Positively selected CD45^+^ cells in the column were retrieved. The number of CD45^+^ cells infiltrating each muscle was correlated with T2-rt.

### Statistics

Data are expressed as means ± standard deviations. Statistical analysis was performed using Student's t-test for unpaired data or one-way ANOVA when appropriate; correlation was evaluated using Pearson test (Prism 4.0, GraphPad Software). Values of p<0.05 were considered statistically significant.

## Results

### MRI assessment of muscle modifications in elder sarcopenia

Sarcopenia refers to the skeletal muscle atrophy and weakness that is associated with aging [14]. We have used multiparametric MRI to detect structural changes associated with sarcopenia in old *vs* young mice (**Table 1**). T2-mapping was performed to investigate edema and DTI to assess anisotropic changes linked to muscle architecture.

**Table 1:** Mice characteristics.

	n	Sex	Age (months)	Weight (g)
Young	8	F	2 ± 0	18.5 ± 0.9
Old	6	F	17.8 ± 2.6	24.8 ± 0.8

Parameters are reported as average ± SD.

**Figure 2:**
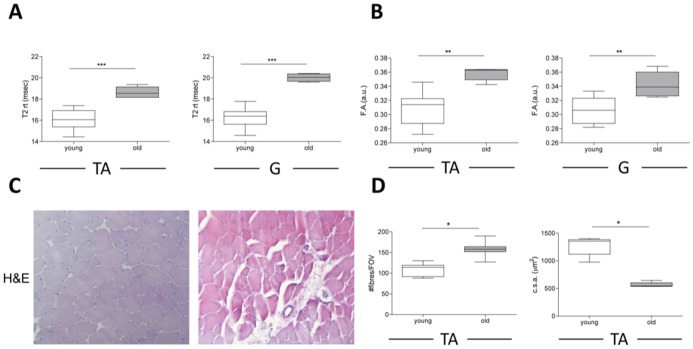
MRI and histological features of skeletal muscles in young and old mice. Analysis of basal levels of T2-rt and F.A. in 8-weeks old C57Bl/6 wt mice (n = 8) and 18 months old C57Bl/6 wt mice (n = 6). A quantitative comparison of T2-rt, F.A. and histology in the two groups of mice is reported for tibialis anterior (TA) muscle (panel A, B and D). Quantitative analysis of T2-rt and F.A. for gastrocnemius (G) muscle is reported in panel A, B. *p<0.05, **p<0.01, ***p<0,001.

Muscles of old mice had higher T2-rt values then those of their younger counterparts (**Figure 2**, panels **A** and **Table 2**). Muscles of vastly different size and architecture such as tibialis anterior and gastrocnemius were similarly affected. Modifications were almost identical in terms of pattern and extent in all the analyzed mice: therefore T2-rt modifications might not reflect local or individual variables, but a common physiological response of the skeletal muscle to aging. We verified the histological correlates of these results, and observed a consistent infiltration of mononucleated cells, scattered in the perifascicular connective tissue (**Figure 2**, panel **C**). Moreover, muscle fibers had irregular shape and size. Only limited fibrosis was detectable. DTI assessment revealed that F.A. was significantly higher in old *vs.* young mice both in tibialis anterior and gastrocnemius muscles (**Figure 2**, panel **B** and **Table 2**). This result probably is related to the histological evidence that the in old mice the size of fibers was smaller while the overall number of fibers per field of vision was higher; this may influence the diffusion of intercellular and intracellular water molecules, resulting in higher F.A. values (**Figure 2**, panel **D**).

**Table 2:** MRI results in young and old mice.

		young	old	*P*
T2 rt (msec)	TA	16.16 ± 0.99	18.89 ± 0.74	0.0008
	G	15.79 ± 0.98	19.59 ± 0.58	2.74*10^-5^
F.A. (a.u.)	TA	0.31 ± 0.02	0.36 ± 0.01	0.001
	G	0.31 ± 0.02	0.34 ± 0.02	0.005

Magnetic resonance parameters of tibialis anterior (TA) and gastrocnemius (G) muscles in young and old mice. Parameters are reported as average ± SD. T2-rt (T2 relaxation time), F.A. (Fractional Anisotropy), a.u. (arbitrary units).

### MRI monitoring during muscle damage and repair

**Figure 3:**
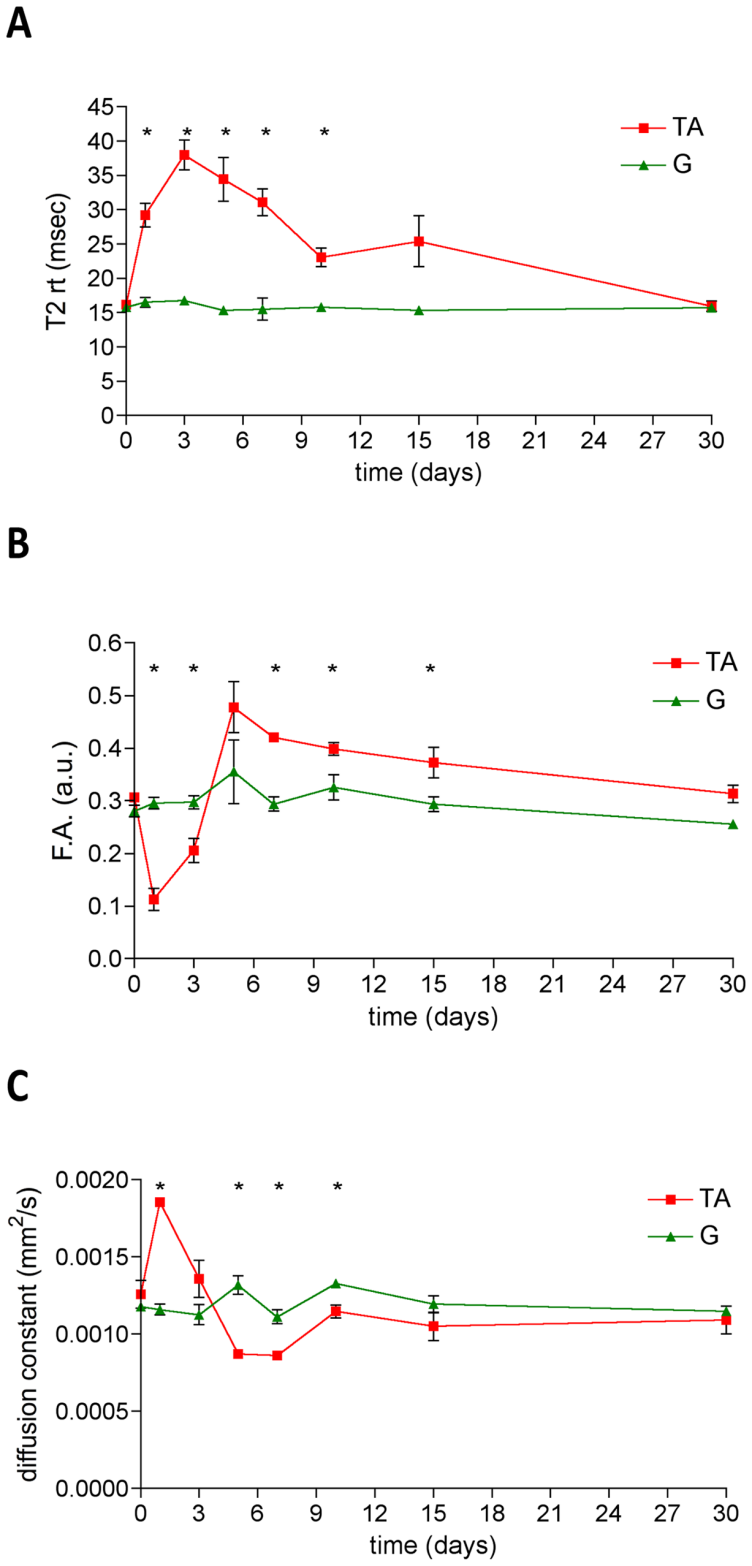
Dynamic changes of MRI parameters during muscle damage and repair. The graphs show the dynamic trends of T2-rt (panel A), F.A. (panel B) and ADC (panel C) in a longitudinal study of C57Bl/6 mice damaged with CTX and followed by MRI before and at day1, 3, 5, 7, 10, 15 and 30 after damage. Red lines indicate tibialis anterior (TA) parameters while green lines represent gastrocnemius (G) parameters. Asterisks (*) label the tibialis anterior parameters that significantly differ from control gastrocnemius muscle parameters. p<0.05 was considered as statistically significant (n = 7 at each time-point).

In order to discriminate the relative contribution of the sequential necrosis, inflammation and regeneration, we studied 23 young mice at various time-points after sterile injury using multiparametric MRI. In particular, the tibialis anterior muscle was damaged with CTX injection and the gastrocnemius muscle was visualized parallel to the damaged one and used in this experimental setting as an internal control of a healthy uninjured muscle. MRI parameters (T2-rt, F.A. and ADC) did not significantly differ between the tibialis anterior and gastrocnemius muscle in basal conditions (day0, **Figure 3** and **Table 3**). T2-rt strongly increased in the tibialis anterior muscle at 24 hours after injury and reached the peak between day3 and day5 after injury. Afterward, a progressive decrease of T2-rt was observed until day30: at this time point, T2-rt returned to basal values (**Figure** **3** **,** panel **A**). Tibialis anterior muscle T2-rt measured at day 1, 3, 5, 7 and 10 after injury was consistently significantly higher than basal values and than T2-rt measured at each time point in the adjacent non-injured gastrocnemius muscle (p<0.05). At later time points, differences progressively abated (**Figure 3** **,** panel **A**). The tibialis anterior F.A. dropped immediately after injury, with the nadir at day1. Afterwards, the F.A. progressively increased, approaching the basal level at day5. From day5 a further slow and progressive F.A. increase was observed, with values significantly higher than the basal F.A. value (**Figures 3** **,** panel **B**). After injury water diffusivity swiftly increased, as documented by a significant diffusion constant increase at day1, both in comparison with basal and adjacent uninjured muscle values. The diffusion constant of the tibialis anterior muscle returned at basal value three days after injury, and then decreased at later time points. At the last time point, when the overall muscle architecture was reconstituted, the tibialis anterior diffusion constant was again comparable with the basal value (**Figure 3** **,** panel **C**).

**Table 3:** MRI parameters variation during muscle damage and repair.

		day0	day1	day3	day5	day7	day10	day15	day30
T2-rt (msec)	TA	16.2±0.8	29.2±3	38.0±3.8	31.1±3.4	31.1±3.4	23.1±2.3	21.7±1.4	15.9±1.3
	G	15.8±1.1	16.5±1.2	16.7±0.9	15.3±0.9	15.5±2.8	15.8±1.8	15.3±1.1	15.7±1.1
	*P*	0.512	0.00001	0.00001	0.00005	0.00002	0.00001	0.012	0.490
F.A. (a.u.)	TA	0.3±0.01	0.1±0.04	0.2±0.04	0.5±0.1	0.4±0.01	0.4±0.02	0.4 ±0.05	0.3 ±0.03
	G	0.28 ±0.02	0.3±0.02	0.3±0.02	0.4±0.1	0.3±0.02	0.3±0.04	0.29±0.02	0.26±0.01
	*P*	0.061	6.44*10^-11^	7.6*10^-4^	0.630	0.005	0.002	4*10^-5^	0.09
ADC (10^-3^mm^2^/s)	TA	1.26±0.02	1.9±0.04	1.36±0.2	0,9±0.02	0.9±0.02	1.2±0.01	1.1±0.16	1.1±0.16
	G	1.18±0.01	1.16±0.01	1.13±0.11	1.32±0.01	1.11±0.01	1.33±0.01	1.2±0.09	1.15±0.04
	*P*	0.139	2.89*10^-6^	0.34	0.003	0.003	0.011	0.28	0.212

Magnetic resonance parameters of tibialis anterior (TA) and gastrocnemius (G) muscles before and after CTX injection (7 mice/time point). Results are reported as average ± SD (*n* = 7). T2-rt (T2 relaxation time), ADC (Apparent Diffusion Coefficient), F.A. (Fractional Anisotropy). *P* refers to comparison between tibialis anterior and gastrocnemius.

**Figure 4:**
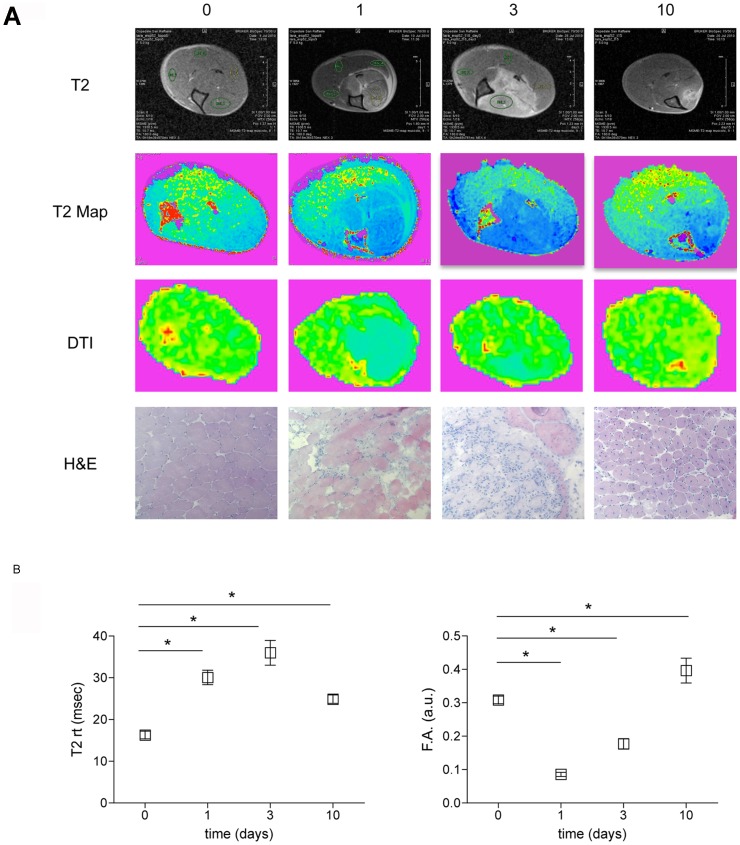
Muscle T2 and FA mapping during damage and repair. Panel A: qualitative comparison between T2-rt map (T2 Map), F.A. map (DTI) and histological findings (H&E) before and at distinct time points after damage. Panel B: The dynamic trends of T2-rt and F.A. in tibialis anterior (TA) muscle are reported. Three animals/time point were studied. Asterisks (*) label the tibialis anterior parameters that significantly differ from basal parameters. p<0.05 were considered as statistically significant.

Before injection (day0) and at day 1, 3, 5, 7, 10, 15, 30 three mice were sacrificed after imaging in order to assess the extent of leukocyte infiltration and the tissue integrity (**Figure 1**). The CTX injection resulted in an early massive and synchronized death of myofibers (day1), followed by substantial infiltration of the tissue by inflammatory cells (prominent at day3). At day10 cell death and inflammation abated and regenerating fibers reconstituted the overall tissue architecture (**Figure 4**, panel **A**). MRI images perfectly paralleled histological findings. T2-rt peaked at day3 resulting significantly higher than at day0 and 10, following tissue changes such as edema and inflammation. The least value of F.A. was achieved at day1 when myofibers were destroyed, while F.A. reached its peak at day10 together with ongoing extensive regeneration, indicating that F.A. may represent a good marker of muscle fibers integrity and regeneration (**Figure 4**, panel **B**).

### T2-rt and F.A. as surrogate markers of tissue inflammation and structural heterogeneity respectively

**Figure 5:**
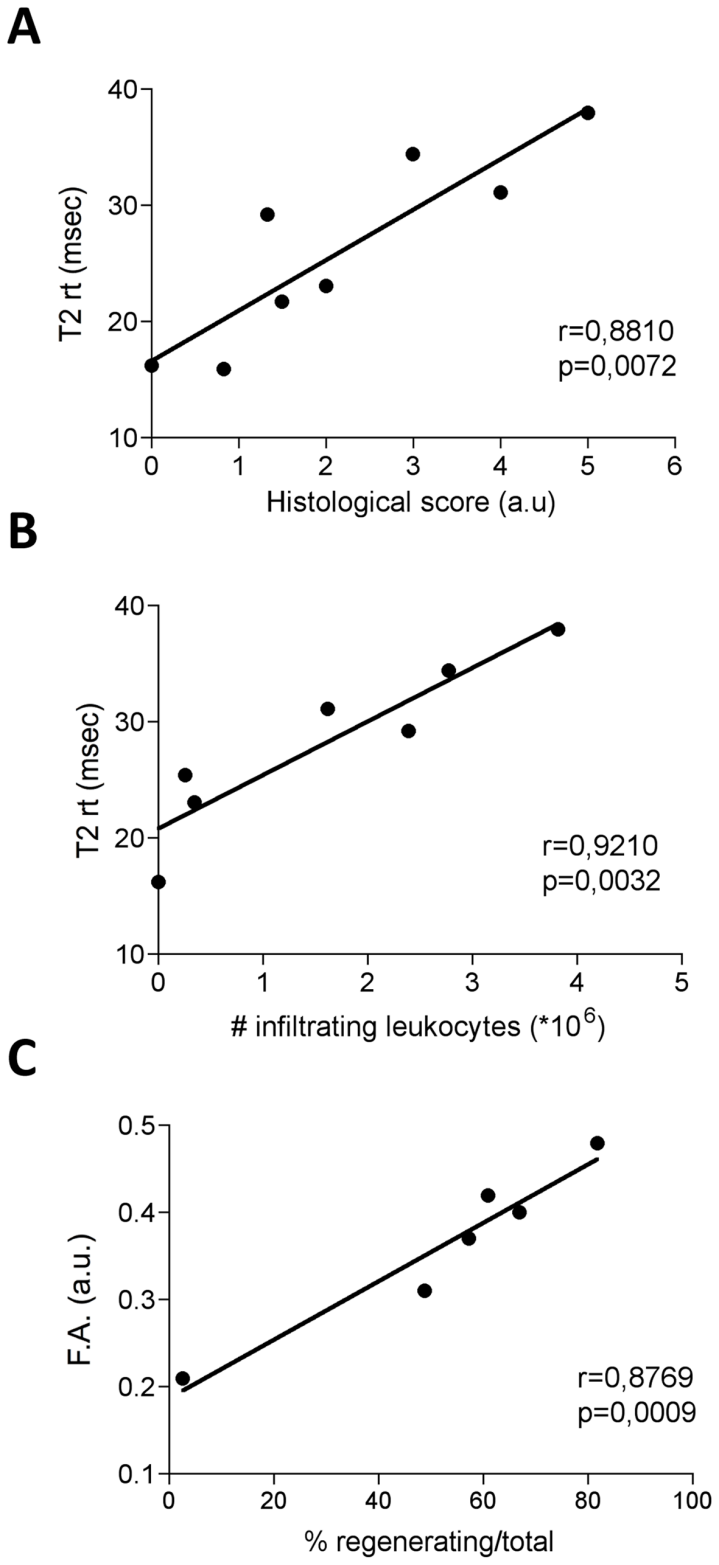
Correlations between MRI and histological changes. The graph in panel A represents correlation analysis between T2-rt and the histological score computing both muscle damage and infiltration (panel A). n = 3 for each time point. The time points considered for the correlation analysis were day 0, 1, 3, 5, 7, 10, 15, 30. The same correlation analysis was performed for T2-rt with the number of infiltrating leukocytes in tibialis anterior (TA) muscle (panel B). Each dot represents the average number of infiltrating leukocytes and the average T2-rt at distinct time points. Time points considered for the correlation analysis were day 0, 1, 3, 5, 7, 10, and 15 after CTX injection. The graph in panel C represents the correlation analysis between F.A. values and the percentage of regenerating fibers in tibialis anterior muscle. Each dot represents the average number of regenerating fibers and F.A. at distinct time points. Time points considered for the correlation analysis were day 3, 5, 7, 10, 15, 30.

The actual link between the values obtained from the T2-map and tissue inflammation was verified assessing the correlation between T2-rt and: *i)* a tissue histological score for inflammation (see the Materials and Methods section for details); *ii)* the extent of tissue infiltration, assessed by the number of CD45^+^ leukocytes retrieved by immuno-magnetic sorting from damaged muscles at various time points after injury. Results of histological assessments and leukocytes numbers are reported in **Table 4**. T2-rt and the histological damage/infiltration score showed a significant correlation (*r* = 0.881; *p* = 0.0072), indicating that T2-rt changes parallel muscle inflammatory infiltration (**Figure 5** **,** panel **A**). An even more impressive correlation was observed between T2-rt and the overall number of CD45^+^ cells retrieved after digestion of the injured tissue (*r* = 0.9210; *p*<0.0032) (**Figure 5** **,** panel **B**), suggesting that T2-rt accurately reflects muscle inflammation.

**Table 4:** Histology results.

	day0	day1	day3	day5	day7	day10	day15	day30
Histological Score (a.u.)	0±0	1.3±0.58	5±0	3.3±0.6	3±0.87	1.9±0.6	1.5±0.9	0.3.±0.4
Infiltrating leukocytes (# CD45^+^ cells * 10^6^ )	0±0	2.39±0.2	3.2±1.7	2.6±0.33	1.6±0.31	0.35±0.07	0.26±0.02	n.a.
Regenerating/total fibers (%)	n.a.	n.a.	2.66±0.15	81.8±13.3	60.9±14	66.9±1.3	57.3±1.44	48.4.±25.3

Markers of damage, infiltration and regeneration evaluated before and after CTX injection in tibialis anterior (TA) muscles (3mice/time point). Results are reported as average ± SD. n.a.: not assessed.

The dramatic drop in F.A. at day1 after injury (**Figures 3B** **and** **4B**) is likely related to fiber necrosis and abrupt loss of fibrillar architecture. In agreement, the F.A. relative rescue observed at day5 was associated to the histological evidence of novel regenerating fibers. The strong correlation between the F.A. values and the fraction of center-nucleated fibers in histological samples (*r* = 0.8769; *p* = 0.0009) supports this interpretation (**Figure 5** **,** panel **C**).

## Discussion

The decline of skeletal muscle mass with aging (an event also referred to as sarcopenia) is the major determinant of age-associated morbidity and mortality [15], [16]. The original view of such event as the consequence of an involutive/degenerative process was challenged by the consistent results of biochemical and histopathological studies, which reveal a role for inflammation in the ability of the tissue to maintain homeostasis, most likely by finely controlling of the muscle stem cell compartment [17], [18], [19], [20]. Hence, a fine interplay between necrosis, inflammation and regeneration physiologically operates in the tissue and is responsible for maintaining its size and function. The actual events taking place in otherwise healthy skeletal muscles in the aging population are however only partially elucidated: this is mainly because of the lack of an effective, non-invasive approach to obtain information on the actual state of the tissue. This possibility would be valuable also in more complex setting of diseases affecting the skeletal muscle, including those related to cancer-related cachexia, diabetes, or autoimmune inflammation [21], [22].

MRI represents a potent and versatile tool for the non-invasive study of the skeletal muscle. The availability of validated information in preclinical settings would be instrumental for making this approach more informative and data interpretation more reliable.

In this study, we have compared the characteristics of the skeletal muscle in otherwise healthy relatively old mice (18 months, *i.e.* an age in which the skeletal muscle mass begins to significantly decline, both absolutely and relatively to the total body mass) [23], to those in healthy young mice. Histological results confirmed that the incipient decline in muscle mass was associated with infiltration by mononuclear cells in all investigated muscles. Moreover variations in the MRI parameters we analyzed, such as T2-rt and F.A., seem to reflect muscle subclinical inflammation and architectural remodeling characterizing muscle aging. In the normal muscle T2-rt is much shorter than in other parenchymas [24], because of the strong contribution of the intracellular muscle compartment in which water T2-rt is very short [25], [26]: thus, from a physical point of view, T2-rt modifications observed in old mice are likely to be substantially influenced by muscle edema and enlargement of the extracellular space, due to the smaller size of the fibers together with the infiltration of the perifascicular connective tissue by mononuclear cells. However, we cannot exclude that a minor part of T2-rt lengthening characterizing muscle aging could be related to other phenomena of age-related muscle remodeling, that were not considered in the present study, such as the increase of adipose tissue content due to skeletal muscle fat infiltration.

To verify in better detail the potential link between the MRI and the histological and histochemical data, we relied on a well-established model of sterile injury, which is characterized by an acute self-limiting inflammatory response, which results in effective and complete regeneration of the tissue. Indeed, we observed that acute muscle necrosis was associated with acute massive self-limiting modifications in MRI parameters. Results obtained by the tight comparison between histology and imaging demonstrated a cogent statistical association between both T2-rt/histological score and T2-rt/number of infiltrating leukocytes. Although, potential confounding factors may participate in the T2-rt dynamic changes induced by sterile injury as, for example, the muscular injection *per se* or the accumulation of necrotic debris in the first phases after injury, the strong relationship found with leukocytes infiltration [2], [3] remarks that T2-rt may represent a good measure of skeletal muscle inflammation in a setting of acute injury. Moreover, the high temporal resolution of our study allowed to observe a distinct modification kinetic of F.A. and T2-rt values. Changes in diffusivity and in T2-rt followed a clearly diverse chronological pattern, in agreement with the hypothesis that they provide information on non-overlapping events occurring during muscle regeneration [3]. As suggested by Damon et al. [27] the diffusion of water measured in skeletal muscles largely reflects the diffusion in the intracellular space. The myocyte swelling and the loss of myocyte integrity, which is constantly detected in histological sections of the tissue early after injury, likely leads to the increase in water diffusion detected 24 hours after injury. Conversely, the progressive substitution of the necrotic material with small regenerating fibers, observed 5 and 7 days after injury, was likely to be involved in the diffusivity restriction observed. The F.A. changes were larger than those observed in water diffusivity. The intact skeletal muscle was characterized by an anisotropic diffusion of water (F.A.≈0.3), which is similar to that of the white matter [1], [3], [28], [29]. F.A. of the skeletal muscle could be influenced by fibers integrity, fibers diameter and fibers density. A loss of fibers integrity is expected to cause a dramatic reduction of F.A.: accordingly, most of the anisotropic structure of the skeletal muscle was lost in the first day after injury. Subsequently, F.A. increased in parallel with the actual regeneration of the tissue and, indeed, between day5 and day7 F.A. was significantly higher than basal values: at these time points most of the necrotic tissue had been substituted by healthy regenerating fibers, which however were still immature and characterized by a smaller size compared to non-injured tissue. Since water diffusion is mainly intracellular and the extracellular space is still largely occupied by infiltrating leukocytes, the small diameter of the newly formed fibers easily justifies the increase of F.A. above the normal level. The further progressive decrease of F.A. toward normal values was accompanied by, and likely due to, progressive fibers maturation, which was histologically labeled by peripheral migration of nuclei and that resulted in an overall larger diameter of fibers. In support of this interpretation, we found a very strong and highly significant correlation between the F.A. and the percentage of regenerating fibers in histological sections.

Changes in T2 and diffusion parameters observed as a response to acute injury were clearly visually perceptible in the parametric maps reported in the Figure 4 (panel A) and T2-rt modifications were also obviously detected observing T2-weighted cross sectional images (first line, panel A, Figure 4). More difficult should be the visual assessment of the more subtle, although significant, changes linked to muscle aging.

The effect of the injection itself was not tested in this study. However, our previous results indicated that the injection of saline *per se* elicits minimal architectural damage or inflammation (not shown). In particular we failed to identify any necrosis or infiltration in the injected tibialis anterior muscle, either immediately after damage or at later time-points, suggesting that the effect of the injection itself is minimal. To minimize the effect of an operator-dependent bias, all evaluations were made independently by blinded pathologists: however the use of histomorphometric analysis to determine fiber c.s.a. and the percentage of regenerating fibers, which by definition involves only a limited and randomly selected areas of a tissue, represents a potential *caveat*. Functional tests could also be valuable to better assess the correlation of structural and inflammatory changes in ageing muscle as detected both by histology and by MRI: in particular studies are on going to verify whether changes of histology and of MRI parameters correlate with treadmill performances of ageing mice, prospectically followed from the two months of age.

Overall, MRI is sensitive not only to relatively strong and acute inflammatory response to the injury: it also allows to non invasively monitor relatively subtle modifications in the architecture and in the inflammatory state of the skeletal muscle, such as those associated with initial age related decline in the muscle mass, which are revealed by the simultaneous increase of T2-rt and F.A.. Further studies are warranted to verify whether specific MRI codes are also associated to other conditions characterized by the persistent inflammation and the wasting of the skeletal muscle.

## References

[1] Heemskerk AM, Strijkers GJ, Vilanova A, Drost MR, Nicolay K (2005) Determination of mouse skeletal muscle architecture using three-dimensional diffusion tensor imaging. Magn Reson Med 53: 1333–1340. 15906281 10.1002/mrm.20476

[2] Heemskerk AM, Sinha TK, Wilson KJ, Ding Z, Damon BM (2009) Quantitative assessment of DTI-based muscle fiber tracking and optimal tracking parameters. Magn Reson Med 61: 467–472. 19161166 10.1002/mrm.21819 PMC2632726

[3] Heemskerk AM, Strijkers GJ, Drost MR, van Bochove GS, Nicolay K (2007) Skeletal muscle degeneration and regeneration after femoral artery ligation in mice: monitoring with diffusion MR imaging. Radiology 243: 413–421. 17384238 10.1148/radiol.2432060491

[4] Fleckenstein JL (1996) Skeletal muscle evaluated by MRI; Grant DMaH, R.K., editor: Chicheseter: Willey.

[5] Loerakker S, Oomens CW, Manders E, Schakel T, Bader DL, et al (2011) Ischemia-reperfusion injury in rat skeletal muscle assessed with T2-weighted and dynamic contrast-enhanced MRI. Magn Reson Med 66: 528–537. 21360588 10.1002/mrm.22801

[6] Marqueste T, Giannesini B, Fur YL, Cozzone PJ, Bendahan D (2008) Comparative MRI analysis of T2 changes associated with single and repeated bouts of downhill running leading to eccentric-induced muscle damage. J Appl Physiol 105: 299–307. 18450983 10.1152/japplphysiol.00738.2007

[7] Mathur S, Vohra RS, Germain SA, Forbes S, Bryant ND, et al (2011) Changes in muscle T2 and tissue damage after downhill running in mdx mice. Muscle Nerve 43: 878–886. 21488051 10.1002/mus.21986 PMC3101319

[8] Wishnia A, Alameddine H, Tardif de Gery S, Leroy-Willig A (2001) Use of magnetic resonance imaging for noninvasive characterization and follow-up of an experimental injury to normal mouse muscles. Neuromuscul Disord 11: 50–55. 11166166 10.1016/s0960-8966(00)00164-4

[9] Collins-Hooper H, Woolley TE, Dyson L, Patel A, Potter P, et al (2012) Age-Related Changes in Speed and Mechanism of Adult Skeletal Muscle Stem Cell Migration. Stem Cell 30: 1182–1195. 10.1002/stem.1088 22593017

[10] Jia Y, Suzuki N, Yamamoto M, Gassmann M, Noguchi CT (2012) Endogenous erythropoietin signaling facilitates skeletal muscle repair and recovery following pharmacologically induced damage. FASEB J 26: 2847–2858. 22490927 10.1096/fj.11-196618 PMC3382092

[11] Julienne CM, Dumas JF, Goupille C, Pinault M, Berri C, et al.. (2012) Cancer cachexia is associated with a decrease in skeletal muscle mitochondrial oxidative capacities without alteration of ATP production efficiency. J Cachexia Sarcopenia Muscle. [Epub ahead of print]. DOI: 10.1007/s13539-012-0071-9. 10.1007/s13539-012-0071-9 PMC3505576 22648737

[12] Holt LJ, Turner N, Mokbel N, Trefely S, Kanzleiter T, et al (2012) Grb10 regulates the development of fiber number in skeletal muscle. FASEB J. 26 (9): 3658–3669. 10.1096/fj.11-199349 22623587

[13] Vezzoli M, Castellani P, Corna G, Castiglioni A, Bosurgi L, et al (2011) High-mobility group box 1 release and redox regulation accompany regeneration and remodeling of skeletal muscle. Antioxid Redox Signal 15: 2161–2174. 21294652 10.1089/ars.2010.3341

[14] Matthews GD, Huang CL, Sun L, Zaidi M (2011) Translational musculoskeletal science: is sarcopenia the next clinical target after osteoporosis? Ann N Y Acad Sci 1237: 95–105. 22082371 10.1111/j.1749-6632.2011.06236.x

[15] Malafarina V, Uriz-Otano F, Iniesta R, Gil-Guerrero L (2012) Sarcopenia in the elderly: diagnosis, physiopathology and treatment. Maturitas 71: 109–114. 22153348 10.1016/j.maturitas.2011.11.012

[16] Beenakker KG, Ling CH, Meskers CG, de Craen AJ, Stijnen T, et al (2010) Patterns of muscle strength loss with age in the general population and patients with a chronic inflammatory state. Ageing Res Rev 9: 431–436. 20553969 10.1016/j.arr.2010.05.005 PMC7105185

[17] Bosurgi L, Manfredi AA, Rovere-Querini P (2011) Macrophages in injured skeletal muscle: a perpetuum mobile causing and limiting fibrosis, prompting or restricting resolution and regeneration. Front Immunol 2: 62. 22566851 10.3389/fimmu.2011.00062 PMC3341990

[18] Paylor B, Natarajan A, Zhang RH, Rossi F (2011) Nonmyogenic cells in skeletal muscle regeneration. Curr Top Dev Biol 96: 139–165. 21621070 10.1016/B978-0-12-385940-2.00006-1

[19] McKay BR, Ogborn DI, Bellamy LM, Tarnopolsky MA, Parise G (2012) Myostatin is associated with age-related human muscle stem cell dysfunction. FASEB J 26: 2509–2521. 22403007 10.1096/fj.11-198663

[20] Armand AS, Laziz I, Djeghloul D, Lecolle S, Bertrand AT, et al (2011) Apoptosis-inducing factor regulates skeletal muscle progenitor cell number and muscle phenotype. PLoS One 6: e27283. 22076146 10.1371/journal.pone.0027283 PMC3208607

[21] Fearon KC (2011) Cancer cachexia and fat-muscle physiology. N Engl J Med 365: 565–567. 21830971 10.1056/NEJMcibr1106880

[22] Mammen AL (2011) Autoimmune myopathies: autoantibodies, phenotypes and pathogenesis. Nat Rev Neurol 7: 343–354. 21654717 10.1038/nrneurol.2011.63

[23] Hamrick MW, Ding KH, Pennington C, Chao YJ, Wu YD, et al (2006) Age-related loss of muscle mass and bone strength in mice is associated with a decline in physical activity and serum leptin. Bone 39: 845–853. 16750436 10.1016/j.bone.2006.04.011

[24] Bottomley PA, Foster TH, Argersinger RE, Pfeifer LM (1984) A review of normal tissue hydrogen NMR relaxation times and relaxation mechanisms from 1-100 MHz: dependence on tissue type, NMR frequency, temperature, species, excision, and age. Med Phys 11: 425–448. 6482839 10.1118/1.595535

[25] Saab G, Thompson RT, Marsh GD (1999) Multicomponent T2 relaxation of in vivo skeletal muscle. Magn Reson Med 42: 150–157. 10398961 10.1002/(sici)1522-2594(199907)42:1<150::aid-mrm20>3.0.co;2-5

[26] Curiel RV, Jones R, Brindle K (2009) Magnetic resonance imaging of the idiopathic inflammatory myopathies: structural and clinical aspects. Ann N Y Acad Sci 1154: 101–114. 19250233 10.1111/j.1749-6632.2009.04386.x

[27] Damon BM, Ding Z, Anderson AW, Freyer AS, Gore JC (2002) Validation of diffusion tensor MRI-based muscle fiber tracking. Magn Reson Med 48: 97–104. 12111936 10.1002/mrm.10198

[28] Schwenzer NF, Steidle G, Martirosian P, Schraml C, Springer F, et al (2009) Diffusion tensor imaging of the human calf muscle: distinct changes in fractional anisotropy and mean diffusion due to passive muscle shortening and stretching. NMR Biomed 22: 1047–1053. 19618408 10.1002/nbm.1409

[29] Saupe N, White LM, Sussman MS, Kassner A, Tomlinson G, et al (2008) Diffusion tensor magnetic resonance imaging of the human calf: comparison between 1.5 T and 3.0 T-preliminary results. Invest Radiol 43: 612–618. 18708854 10.1097/RLI.0b013e31817e909f

